# Ethyl-acetate fraction from a cinnamon-cortex extract protects pancreatic β-cells from oxidative stress damage

**DOI:** 10.3389/fphar.2023.1111860

**Published:** 2023-03-06

**Authors:** Weiling Li, Jialu Qiao, Kuan Lin, Ping Sun, Yuansong Wang, Qian Peng, Xiansheng Ye, Wei Liu, Binlian Sun

**Affiliations:** School of Medicine, Wuhan Institute of Biomedical Sciences, Jianghan University, Wuhan, China

**Keywords:** PDX-1, antioxidant activity, apoptosis, pancreatic β cell, cinnamon cortex

## Abstract

**Background:** The pathogenesis of diabetes mellitus is mediated mainly by oxidative stress produced by damaged pancreatic β-cells. We identified that an ethyl-acetate fraction (EA) from a cinnamon-cortex extract (CCE) is rich in flavonoid, and showed no toxicity to β cells.

**Objective:** In this study, we evaluated the pharmacologic activities of EA on pancreatic β cells using a model of oxidative stress induced by H_2_O_2_ or alloxan.

**Results:** The results showed that EA could significantly reduce reactive oxygen (ROS) accumulation to improve the survival of cells. Western blot showed that EA treatment upregulated expression of nuclear factor erythroid 2 related factor 2, heme oxygenase-1, and gamma glutamylcysteine synthetase. The same model study found that EA also can protect β cells against the apoptosis induced by oxidative stress. Furthermore, EA can enhance insulin secretion in rat and mouse β cell lines treated or not with alloxan or H_2_O_2_. The expression of the insulin transcription factor PDX-1 increased in an EA concentration-dependent manner. At last, the major functional compounds of EA analysis showed that three compounds, cinnamyl alcohol, coumarin, and cinnamic acid, had similar effects as EA.

**Conclusions:** In sum, our data suggested that EA fraction from CCE can protect β cells from oxidative stress, and increase insulin secretion to improve the function of β cells. This function might be due to these three compounds found in EA. Our findings provide a theoretical basis and functional molecules for the use of CCE against diabetes mellitus.

## 1 Introduction

Diabetes mellitus (DM) is a metabolic disease which results in uncontrolled high blood sugar. The prevalence of DM is increasing rapidly worldwide (Demir et al., 2021). Long-term hyperglycemia leads to the damage and dysfunction of various organs, which seriously affects the health and quality of life of patients. Hyperglycemic-induced ROS production (composed mostly of H_2_O_2_ and O_2_^−^) takes part in DM developing and associated complications (Black, 2022). When the levels of ROS exceed the cell’s ability to detoxify it, the equilibrium is breached, then the cell enters into oxidative stress (Oguntibeju, 2019). One of the major mechanisms for DM is that high levels of ROS, which can induce β cell apoptosis, reduce β cell proliferation, and damage β cell function (Mathis et al., 2001; Baumel-Alterzon and Scott, 2022). Despite the advent of numerous anti-hyperglycemic agents, there are significant challenges in optimizing therapies and reducing the side-effects associated with them. Recently, extensive studies have been conducted on antioxidants to reduce the excessive production of ROS observed in DM (Darenskaya et al., 2021). Natural antioxidants showed *in vitro* and *in vivo* protective effects on the recovery and preservation of functional β cells (Zhang et al., 2020). These studies encourage further investigation on the beneficial effects of natural compounds in diabetic condition.

Cinnamon is used as a spice worldwide. Aqueous or ethanol extracts of cinnamon have been shown to exert anti-DM effects in several preclinical and clinical investigations (Mang et al., 2006; Qureshi et al., 2019; Zhou et al., 2022). Diverse species of cinnamon have been reported to have different hypoglycemic effects (Lu et al., 2011), but their constituents and underlying molecular mechanism of action are incompletely understood. Studies have reported that cinnamon extracts can inhibit α-amylase and α-glucosidase activities (Adisakwattana et al., 2011; Mohamed Sham Shihabudeen et al., 2011; Hayward et al., 2019). The monomer procyanidin C1 and cinnamaldehyde isolated from cinnamon have been reported to enhance insulin sensitivity (Li et al., 2015; Sun et al., 2019). Several types of compounds, including flavonoids and phenylpropanoids, have been identified from cinnamon (Farag et al., 2022). It was proven that flavonoids have a crucial role in DM, hyperlipidemia, and neurodegenerative diseases (Al-Ishaq et al., 2019; Hussain et al., 2020). Recently phenylpropanoids have been reported to prevent post-prandial hyperglycemia and type 2 diabetes mellitus by promoting glucose uptake (Yoshioka et al., 2022). Studies have illustrated several benefits of specific dietary natural antioxidants on DM: reducing apoptosis, promoting proliferation of β cells, alleviating oxidative stress, inhibiting α-glucosidase activity, and increasing insulin production (Babu et al., 2013; Wang et al., 2019; El-Ouady et al., 2021) [17–19]. Therefore, antioxidants from cinnamon extract could be developed into drugs used for DM management.

We wished to evaluate the anti-DM effect of an ethyl-acetate fraction (EA) from a cinnamon-cortex extract (CCE) on damaged pancreatic β cells treated with alloxan or H_2_O_2_. We found that β cells could be protected from damage and activated to produce insulin after EA treatment. Then, we identified the major functional compounds present in EA. These findings provide a theoretical basis for therapy of DM using cinnamon.

## 2 Materials and methods

### 2.1 Preparation of CCE extract

CCE was prepared from ground cinnamon purchased from a local pharmacy. Briefly, cinnamon powder (100 g) was extracted with 60% aqueous ethanol (1 L) for 1.5 h at 70°C. The aqueous portion was partitioned by ethyl acetate to obtain EA. The concentrated solutions were adsorbed onto AB-8 macro porous resin (Cohesion, Beijing, China). Furthermore, water and ethanol were used to remove excess carbohydrates and low-molecular-weight compounds. The solution was eluted with 80% ethanol and then concentrated and freeze-dried into powder to obtain purified EA. The total content of flavonoids and phenolic compounds was determined as milligrams of rutin equivalents (RE) and gallic-acid equivalents (GAE), respectively, per gram of extract. The chemical composition of EA was examined using high-performance liquid chromatography (HPLC) employing a 1,260 Infinity system (Agilent Technologies, USA) with an ultraviolet detector. The sample was separated using a C18 column (4.6 × 250 mm, 5 μm; PerkinElmer, USA), and analyzed using a mobile phase of 0.3% acetic acid in water and acetonitrile. Absorbance was measured at 254 nm and 280 nm.

### 2.2 Evaluation of the antioxidant capacity of EA *in vitro*

We conducted assays to measure the ability to scavenge radicals (hydroxyl and superoxide). These assays provided a basis for further research on the antioxidant capacity of EA from CCE. For the assays, EA and vitamin C (VC) of different concentrations (0.1, 0.25, 0.5, 1 mg/mL) were prepared.

#### 2.2.1 Hydroxyl radical scavenging assay

The test of the ability to scavenge hydroxyl radicals was carried out according to the method described by the previous study with minor modifications (Zhang et al., 2014). A mixture of solutions containing FeSO_4_ (9 mM, 100 μL), H_2_O_2_ (6 mM, 2 mL), and test sample (2 mL) were incubated for 10 min at 25°C. Then, salicylic acid (9 mM, 2 mL) was dripped into the mixture, followed by incubation for 30 min at 25°C. Absorbance was measured at 518 nm. Percent scavenging inhibition (%) was calculated using the following equation:

$$Scavenging\;of\;hydroxyl\;radicals\;\left(\%\right)=\left[\left(A0-A1\right)/\left(A2-A1\right)\right]\times 100$$

where A0, A1, and A2 are the absorbance of the sample, blank, and control, respectively.

#### 2.2.2 Superoxide radical scavenging assay

The test of the ability to scavenge superoxide radicals was carried out according to the method described by the previous study with minor modifications (Li, 2012). Briefly, the sample (1 mL) was introduced into a test tube containing 50 mM Tris-HCl buffer (pH 8.2, 4.5 mL) and 25 mM pyrogallol (0.4 mL) followed by incubation for 5 min at 25°C. Then, HCl (8 mM, 1 mL) was added to the mixture. Absorbance was measured at 420 nm. The scavenging inhibition (%) was calculated using the equation shown above.

### 2.3 Cell culture

The reagents used in cell culture were purchased from Gibco. An insulinoma cell line (INS-1) from rats was cultured in RPMI-1640 medium. A pancreatic beta cell line (MIN6) from mice was grown in high-glucose Dulbecco’s modified Eagle’s medium (DMEM) containing 10% fetal bovine serum, 1% penicillin–streptomycin, and 0.2% β-mercaptoethanol. Cells were maintained at 37°C in an atmosphere of 5% CO_2_ in humidified air. To establish a model of oxidative stress in the β cell, INS-1 cells were cultured with alloxan (18 mM; Sigma, United States) or MIN6 cells were cultured with H_2_O_2_ (150 μM; Sigma) for 24h, which formed the oxidative stress (OS) model groups (Kimoto et al., 2003; Li et al., 2020).

A stock solution of EA (40 mg/mL) was prepared. The main ingredients coumarin, cinnamyl alcohol, cinnamic acid, cinnamaldehyde (purity: HPLC ≥98%) were purchased from Shanghai Yuanye Bio-Technology (Shanghai, China) and prepared as stock solutions (50 mM) and diluted to a final concentration of 50 µM. The concentration required for experimentation was prepared with medium immediately before use.

### 2.4 Measurement of cell viability

Cell Counting Kit-8 (CCK-8; Beyotime Institute of Biotechnology, Shanghai, China) was used to determine the toxicity and protection of EA on INS-1 cells or MIN6 cells. INS-1 cells or MIN6 cells were seeded into 96-well plates at 5 × 10^4^ cells/well and cultured overnight. Then, they were treated/not treated with alloxan/H_2_O_2_, and continued to be exposed to various concentrations of EA for 24 h. Then, CCK-8 solution (10 μL) was added. After incubation for 1 h at 37°C, absorbance was measured at 450 nm with a microplate reader. Each experiment was replicated in six wells. Cell viability was presented as a percentage of that of the control.

Living cells can be stained with calcein-AM (Beyotime Institute of Biotechnology) but dead cells cannot. We rinsed cells with phosphate-buffered saline (PBS) and incubated them with calcein-AM (2 μM) for 30 min at 37°C. Images of cells were acquired with a fluorescence microscope (IX51 series; Olympus, Tokyo, Japan).

### 2.5 Biochemical analyses

A Nitric Oxide Assay Kit (Beyotime Institute of Biotechnology) was used to quantify nitric oxide (NO) release according to manufacturer protocols. Briefly, standards of NaNO_2_ (50 μL; 0, 1, 2, 5, 10, 20, 40, 60, 100 μM) or cell-culture supernatants (50 μL) were dispensed into a 96-well plate. Then, Griess reagent I (50 μL) and Griess reagent II (50 μL) were added. After incubation for 3 min, absorbance was measured at 540 nm. NO production was calculated according to the standard curve and expressed as a percentage of that of the control.

Commercial kits were used to assay for superoxide dismutase (SOD) activity (Beyotime Institute of Biotechnology), reduced glutathione (GSH) content (microplate method; Nanjing Jiancheng Bioengineering Institute, Nanjing, China), and malondialdehyde (MDA) content (colorimetric method; Nanjing Jiancheng Bioengineering Institute, Nanjing, China). Briefly, INS-1 cells or MIN6 cells were seeded at 1×10^6^ cells/well in six-well plates and cultured overnight. After the indicated treatment, cells were washed twice with PBS, resuspended with PBS, sonicated on ice and centrifuged for 15 min at 4°C. Supernatants were used to measure the activity of SOD as well as levels of GSH and MDA. Finally, data were obtained under different wavelengths (532, 420, 405 nm) using a plate reader. Results were calculated with reference to a standard curve and expressed as nmol/mg protein for MDA, μmol/mg protein for GSH, and U/mg protein for SOD.

### 2.6 ROS determination

The fluorescent probe 2′,7′-dichlorofluoresceindiacetate (DCFH-DA, Sigma-Aldrich, St. Louis, MO) was used to measure the ROS level. INS-1 cells or MIN6 cells were seeded 1 × 10^6^ cells/well in six-well plates and cultured overnight. After the indicated treatment, cells were washed with Hank’s balanced salt solution (HBSS) and incubated with DCFH-DA (10 μM) for 30 min at 37°C in the dark. The fluorescence absorption of DCF was measured using a microplate reader (Fluoroskan™; Thermo Fisher Scientific, USA) at an excitation wavelength of 485 nm and emission wavelength of 538 nm. The fluorescence intensity was proportional to the amount of ROS generated intracellularly.

### 2.7 Flow cytometry

An Annexin V-FITC Apoptosis Detection Kit (Beyotime Institute of Biotechnology) was used to quantify apoptosis according to standard procedures. Briefly, after the indicated treatment, binding buffer (195 μL) containing annexin V-fluorescein isothiocyanate (5 μL) and propidium iodide (10 μL) was added to a single-cell suspension and the reaction allowed to proceed for 15 min at room temperature in the dark. Then, percent apoptosis was evaluated by flow cytometry using a C6 Plus system (BD Biosciences, United States). A minimum of 10,000 cells were detected for each sample.

### 2.8 Caspase activity assay

The activity of caspase-3 and caspase-9 was assessed using the corresponding Caspase Activity Assay Kit (Beyotime Institute of Biotechnology). In brief, cells in a six-well plate underwent the indicated treatment, were washed in PBS, suspended in lysis buffer (150 μL; provided in the kit) and centrifuged for 15 min at 4°C. The supernatant was harvested and mixed with peptide substrate (Ac-DEVD-pNA for caspase-3 and Ac-LEHD-pNA for caspase-9). Absorbance was measured using a microplate reader at 405 nm.

### 2.9 Western blotting

Proteins were extracted using RIPA buffer (Beyotime Institute of Biotechnology) followed by addition of a fresh protease-inhibitor cocktail and phenylmethylsulfonyl fluoride. The protein concentration was determined using a BCA Protein Assay Kit (Boster Biological Technology, China). Proteins (30 μg) were separated by sodium dodecyl sulfate–polyacrylamide gel electrophoresis using 10% gels, followed by transfer to polyvinylidene fluoride (PVDF) membranes (Millipore, USA). After blockade with 5% non-fat milk in Tris-buffered saline–Tween buffer, the blot was probed with target-specific primary antibodies. The primary antibodies we used were those against nuclear factor erythroid 2 related factor 2 (Nrf2; catalog number: abs130481; Absin Bioscience, Shanghai, China), heme oxygenase 1 (HO-1; abs131494; Absin Bioscience), gamma glutamylcysteine synthetase (γ-GCSc; abs138070; Absin Bioscience), inducible nitric oxide synthase (iNOS; 22226-1-AP; Proteintech, United States), cleaved caspase-3 (9661S; Cell Signaling Technology, United States), Bax (AF1270; Beyotime Institute of Biotechnology), glyceraldehyde 3-phosphate dehydrogenase (GAPDH; 60004-1-Ig; Proteintech), and lamin B1 (abs155437; Absin Bioscience). Subsequently, PVDF membranes were incubated with the appropriate horseradish peroxidase-conjugated secondary antibody (Boster Biological Technology). Target proteins were visualized with BeyoECL Moon (Beyotime Institute of Biotechnology) using the Chemidoc XRS Gel Imaging System (Bio-Rad Laboratories, United States).

### 2.10 Insulin secretion assay

INS-1 cells or MIN6 cells were seeded at 1 × 10^5^ cells/well in 48-well plates, followed by treatment/non-treatment with EA or alloxan/H_2_O_2_ for 24 h. Then, cells were incubated in HBSS containing 0.2% bovine serum albumin for 2 h. Then, the buffer was changed to HBSS containing basal glucose (5.5 mM) or glucose (5.5 mM) plus KCl (30 mM) followed by incubation for 1 h, respectively. The insulin content in the culture supernatant was measured by an Insulin ELISA Kit for rat or mice (Bio-Swamp, Wuhan, China).

### 2.11 Quantitative real-time PCR analysis

TRIzol^®^ Reagent was employed to purify the total RNA obtained from cells according to manufacturer instructions (Invitrogen, Carlsbad, CA, United States). RNA and (2 μg) was used to synthesize complimentary (c) DNA using the M-MLV Reverse Transcriptase Kit (Takara Biotechnology, Shiga, Japan). The obtained cDNAs were employed as templates to determine mRNA expression of the insulin transcription factor PDX-1 by real-time RT-qPCR using the TB Green^®^ Premix Ex Taq™ Kit (Takara Biotechnology). Amplification was carried out using a thermocycler (CFX 96; Bio-Rad Laboratories). The primers for PDX-1 were 5′-CAA​AGC​TCA​CGC​GTG​GAA​AA-3′ (sense) and 5′-CGA​GGT​TAC​GGC​ACA​ATC​CT-3′ (antisense). The primers for β-actin were 5′-CAC​CCG​CGA​GTA​CAA​CCT​TC-3′ (sense) and 5′- CCC​ATA​CCC​ACC​CAT​CAC​ACC-3′ (antisense). Relative expression of PDX-1 was calculated using the 2^−ΔΔCT^ method.

### 2.12 Statistical analyses

All assays were carried out in triplicate. Measurement data are the mean ± SEM. The Student’s t-test or one-way ANOVA was conducted to estimate significant differences using Prism 5 (GraphPad, La Jolla, CA, United States), *p* < 0.05 was considered significant.

## 3 Results

### 3.1 EA fraction from CCE is rich in flavonoids

We obtained EA fraction from CCE. Then, we determined the total flavonoid content and phenol content of EA: 89.93 ± 7.96 mg per gram RE equivalent and 34.26 + 0.78 mg per gram GAE equivalent (Table 1), which indicated that the total flavonoid content of the EA extract was higher than the total phenol content. Chemical assays were used to detect the antioxidant capacity of EA. The inhibition percent radical scavenging of EA indicated that EA containing abundant flavonoids had antioxidant capacity.

**TABLE 1:** Phenolic content, flavonoid content, and radical-scavenging assays of the ethyl-acetate fraction from the cinnamon cortex extract.

Sample	Phenolic (mg GAE/g)	Flavonoid (mg RE/g)	Hydroxyl inhibition (%)	Superoxide anion inhibition (%)
EA	34.26 + 0.78	89.93 + 7.96	40.6 + 1.1	68.1 + 2.0
VC	—	—	50.5 + 0.7	71.8 + 0.9

Values are the mean ± SEM, of two determinations; RE, rutin equivalent; GAE, gallic acid equivalent; VC, acted as a positive control. The scavenging capacity was detected at the concentration of 1.0 mg/mL.

### 3.2 Protective effects of EA on β cell lines

**FIGURE 1:**
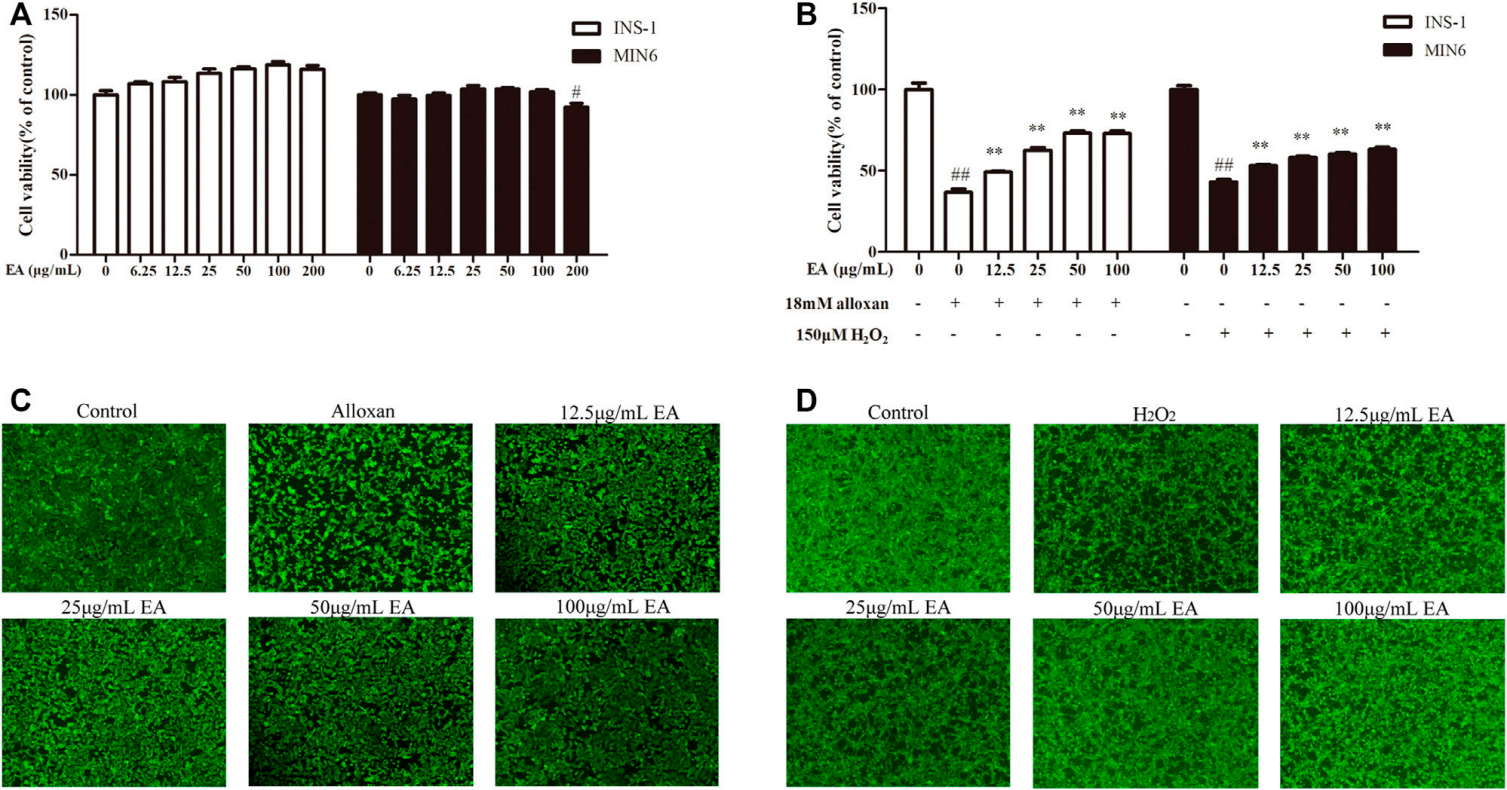
Cytotoxicity and cytoprotective effects of EA in β cell lines. INS-1 cells and MIN6 cells were exposed to EA (0–200 μg/mL) for 24 h **(A)**, INS-1 cells were co-treated with the indicated concentrations of EA and alloxan (18 mM), or MIN6 cells were co-treated with the indicated concentrations of EA and H_2_O_2_ (150 μM) for 24 h, respectively **(B)**, and then the cell viability was determined by the CCK-8 assay. **(C,D)** Morphologic observation of INS-1 cells and MIN6 cells treated as indicated and stained with calcein-AM. Representative images were acquired by a fluorescence microscope. Treatment of cells with EA markedly improved the morphologic changes of INS-1 cells caused by alloxan and in MIN6 cells caused by H_2_O_2_. Data are the mean ± SEM (n = 3). ^#^ *p* < 0.05 compared with control, ^##^ *p* < 0.01 compared with control, *p < 0.05 compared with the OS-model group, **p < 0.01 compared with the OS-model group.

To determine the possible cytotoxicity of EA, cell viability was determined using the CCK-8 assay. INS-1 cells and MIN6 cells were incubated with EA (0–200 μg/mL) for 24 h. The CCK8 assay showed that EA did not cause cytotoxicity up to 100 μg/mL (Figure 1A). It has been reported that treatment with alloxan (18 mM) or H_2_O_2_ (150 μM) can induce β cells damage (Kimoto et al., 2003; Li et al., 2020). Hence, to evaluate if EA exerted protective effects upon β cells, we co-treated INS-1 cells or MIN6 cells with alloxan/H_2_O_2_ and EA. The proliferation of INS-1 cells was enhanced to 12.5%, 25.8%, 36.5%, and 36.25% at concentrations of 12.5, 25, 50, and 100 μg/mL of EA, respectively, compared with that in the OS-model group (Figure 1B). For MIN6 cells treated with H_2_O_2_, the viability was enhanced to 10.2%, 15.1%, 17.2%, and 20.2% at concentrations of 12.5, 25, 50, and 100 μg/mL of EA, respectively, compared with that in the OS-model group. Similarly, stimulation with alloxan/H_2_O_2_ in β cells resulted in a reduced mean fluorescence intensity of calcein-AM, which was reversed by treatment with EA (Figures 1C, D), which indicated that EA could increase β cells viability. These results indicated that EA was not toxic to β cells but also protected them from the damage induced by alloxan or H_2_O_2_.

### 3.3 Protective effects of EA against oxidative stress in β cells

**FIGURE 2:**
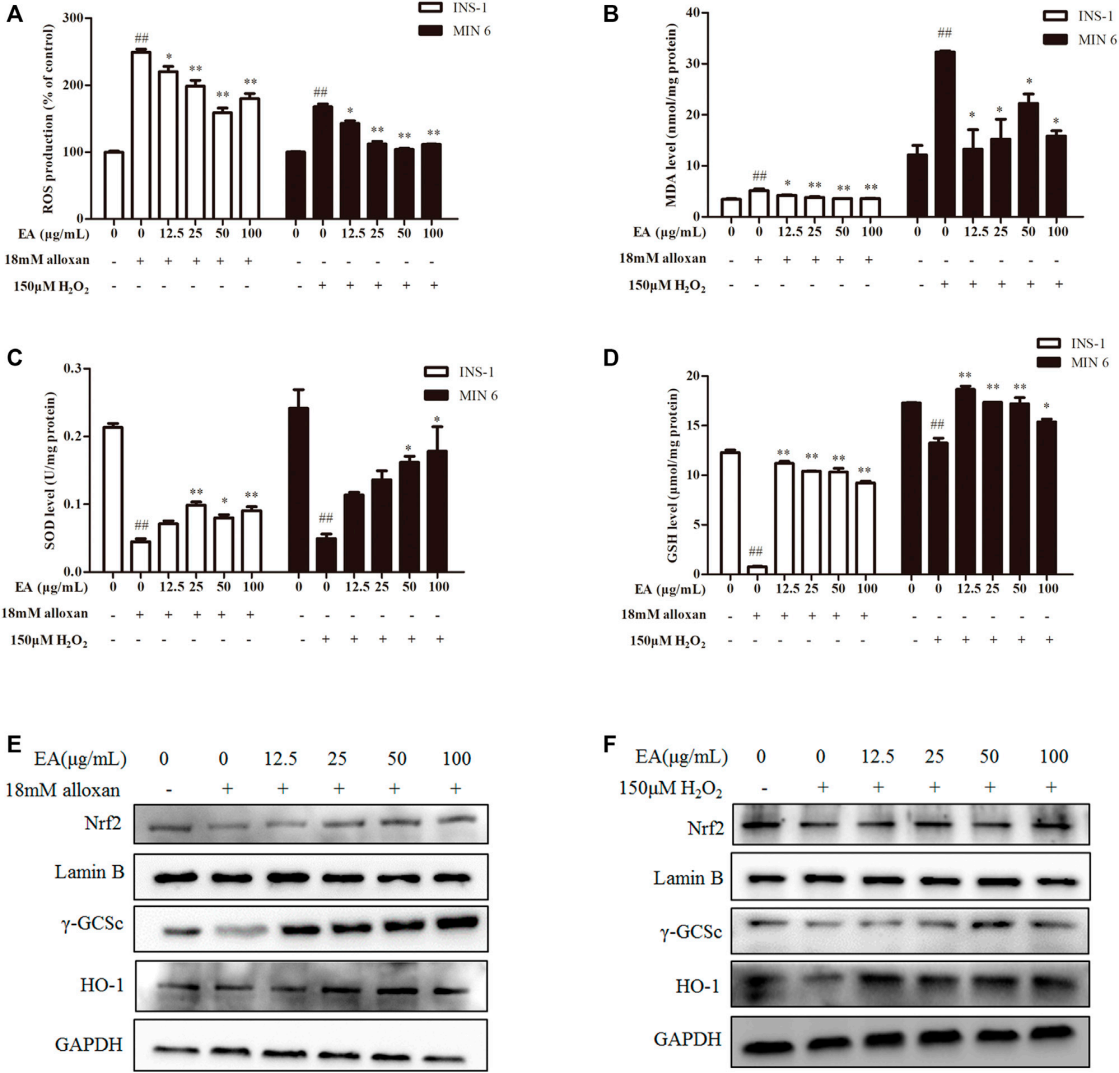
EA protects β cells from oxidative stress damage. INS-1 cells were incubated with EA (12.5–100 μg/mL) and alloxan for 24 h, MIN6 cells were incubated with EA (12.5–100 μg/mL) and H_2_O_2_ for 24 h. Levels of oxidative stress-related makers were determined. **(A)** Analysis of the mean fluorescence intensity of DCF (indicator of reactive oxygen species). Effect of EA on MDA production **(B)**, SOD activity **(C)**, and on the level of reduced GSH **(D)** in oxidative stress-treated β cells. **(E,F)** Protein expression of Nrf 2, γ-GCSc, and HO-1 of alloxan (18 mM)-treated cells **(E)** and H_2_O_2_ (150 μM)-treated cells **(F)** was measured by western blotting. Data are the mean ± SEM (n = 3). ^#^ *p* < 0.05 compared with control, ^##^ *p* < 0.01 compared with control, *p < 0.05 compared with the OS-model group, **p < 0.01 compared with the OS-model group.

Studies have demonstrated that oxidative stress caused by increased ROS generation is the primary cause of β cell damage (Dinić et al., 2022). We wondered if EA affects ROS production under alloxan/H_2_O_2_ treatment. We detected the effect of EA on ROS production with the fluorescent probe DCFH-DA in the β cells co-treated with EA and alloxan or H_2_O_2_ for 24 h. EA suppressed ROS production significantly (Figure 2A). The antioxidant capacity of EA was evaluated by measuring the activity or level of markers of ROS-mediated injury: MDA, SOD, and GSH. In the β cells co-treated with EA, and alloxan or H_2_O_2_ for 24 h, the MDA level was suppressed, and SOD activity and GSH content recovered, by dose-dependent treatment with EA. These data implied that EA treatment improved endogenous antioxidant activities and inhibited lipid peroxidation significantly (*p* < 0.05 vs. OS-model group) (Figures 2B–D). These findings suggested that EA appeared to have a protective action *via* the improvement of antioxidant capacities and reducing oxidative stress by suppressing ROS and MDA production in β cells.

Nrf2/HO-1 is a vital signaling pathway that regulates oxidative stress in cells. We wished to ascertain whether the antioxidant effect of EA correlated with regulation of the Nrf2/HO-1 signaling pathway. Hence, we measured expression of Nrf2 and its downstream proteins (HO-1, γ-GCSc) in INS-1 cells and MIN6 cells co-treated with alloxan or H_2_O_2_ and the indicated EA concentration. Western blotting showed that protein expression of Nrf2, γ-GCSc, and HO-1 was decreased in alloxan- or H_2_O_2-_treated cells, but was increased significantly under EA treatment (Figures 2E, F). These results implied that EA could suppress the oxidative stresses induced by alloxan/H_2_O_2_ by activating the Nrf2/HO-1 pathway.

### 3.4 EA protects β cells from apoptosis

**FIGURE 3:**
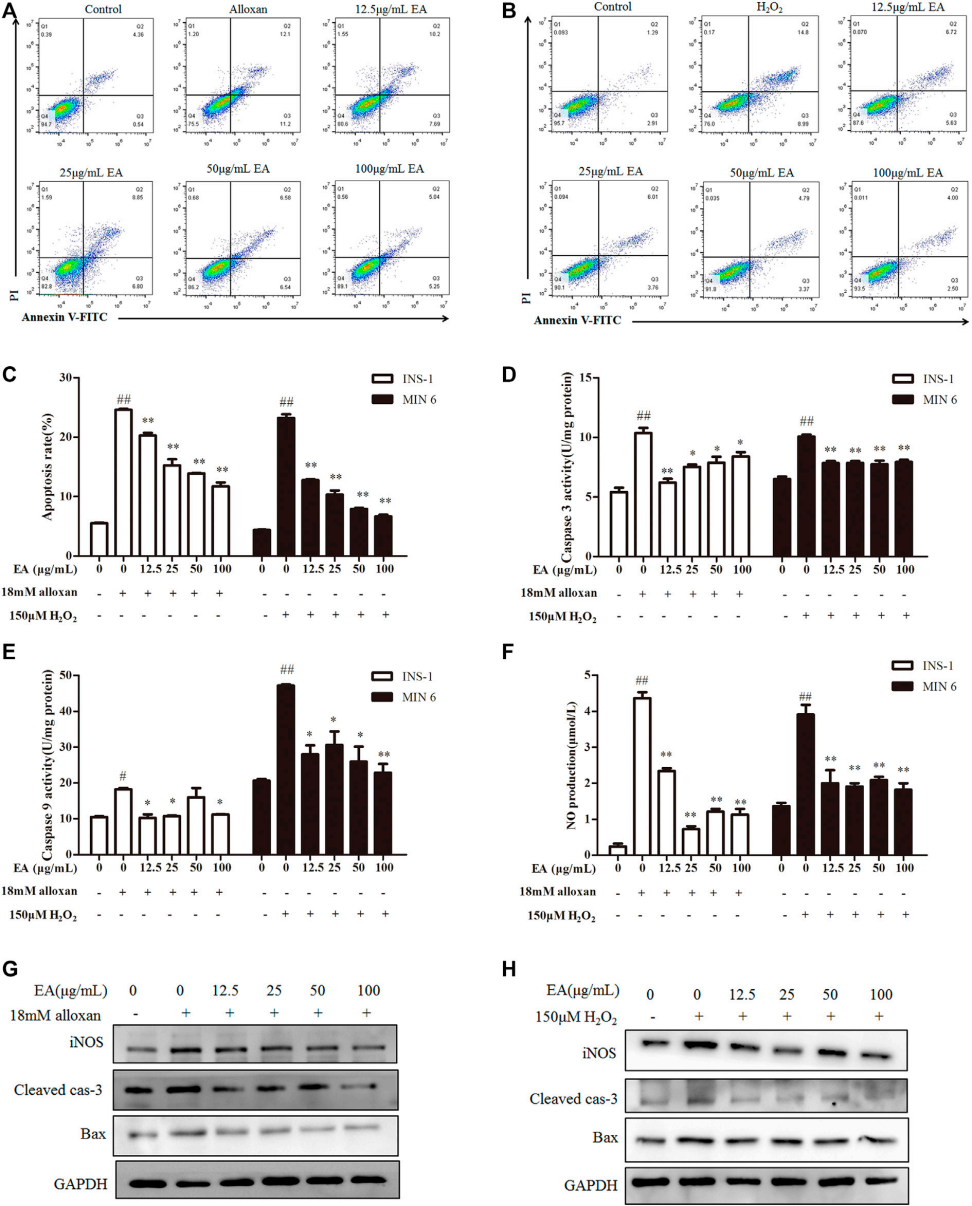
Effect of EA on apoptosis of β cells. INS-1 cells were incubated with EA (12.5–100 μg/mL) and alloxan for 24 h. MIN6 cells were incubated with EA (12.5–100 μg/mL) and H_2_O_2_ for 24 h. Levels of apoptosis-related makers were determined. **(A–C)** Effect of EA on apoptosis was detected with flow cytometry, **(D,E)** Effect of EA on the activity of caspase-3 and caspase-9. **(F)** Effect of EA on NO release into the supernatant. **(G,H)** Western blots showing protein expression of iNOS, Bax, and cleaved caspase-3. Data are the mean ± SEM, n = 3. ^#^ *p* < 0.05 compared with control, ^##^ *p* < 0.01 compared with control, *p < 0.05 compared with the OS-model group, **p < 0.01 compared with the OS-model group.

Apoptosis is an important result of OS. Hence, we investigated if EA could suppress oxidative stress-induced apoptosis. Flow cytometry showed that percent apoptosis of β cells was increased significantly under treatment with alloxan or H_2_O_2_ (Figures 3A, B). However, the addition of EA reduced the number of apoptotic cells markedly. To confirm these results, we measured the activity of caspase-3 and caspase-9 in treated cells: EA reversed the higher activity induced by treatment with alloxan or H_2_O_2_ (Figures 3C, D). Excessive production of NO is regarded as one of the critical molecular mechanisms leading to apoptosis of pancreatic β cells (Oyadomari et al., 2001). EA treatment reduced NO production in pancreatic β cells induced by treatment with alloxan or H_2_O_2_ (Figure 3F). NO release and apoptosis induction were further confirmed by protein expression of iNOS, cleaved caspase-3, and Bax by western blotting. Addition of EA (12.5–100 μg/mL) could suppress expression of iNOS, cleaved caspase-3, and Bax induced by treatment with alloxan or H_2_O_2_ (Figures 3G, H). Collectively, these data suggested that EA had protective effects against the apoptosis of pancreatic β cells induced by oxidative stress.

### 3.5 EA treatment enhances insulin secretion of β cells

**FIGURE 4:**
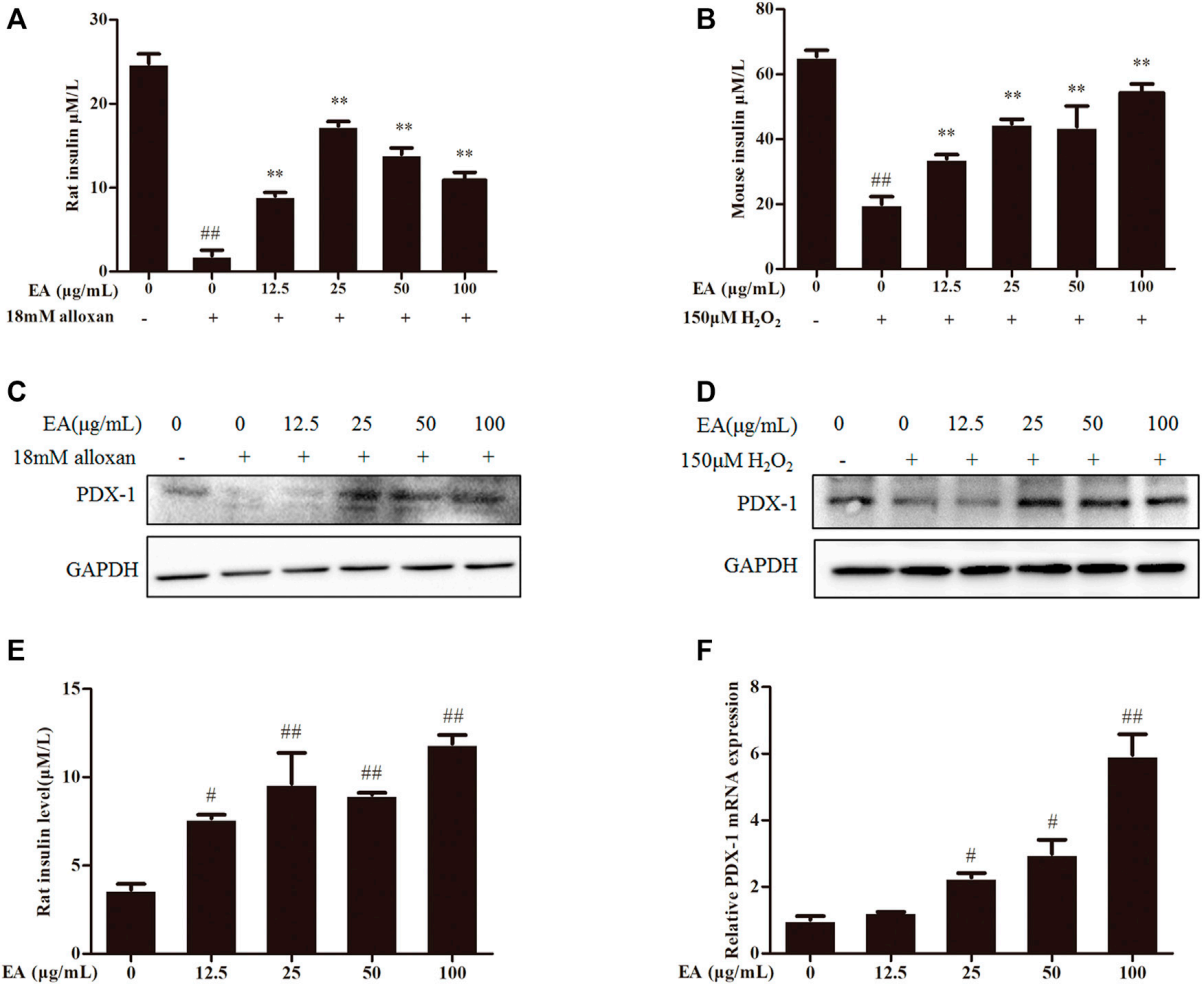
EA increases insulin secretion *via* enhancing PDX-1 expression. INS-1 cells were incubated with EA (12.5–100 μg/mL) and alloxan for 24 h. MIN6 cells were incubated with EA (12.5–100 μg/mL) and H_2_O_2_ for 24 h **(A,B)** Effect of EA on KCl-stimulated insulin secretion in INS-1 cells and MIN6 cells under oxidative stress-induced toxicity was detected using ELISA. **(C,D)** Effect of EA on protein expression of PDX-1 in INS-1 cells and MIN6 cells under oxidative stress-induced toxicity was detected by western blotting. **(E)** Effect of EA on basal glucose-stimulated insulin secretion in INS-1 cells was determined by ELISA. **(F)** Effect of EA on mRNA expression of PDX-1 in INS-1 cells was detected by qPCR. Data are the mean ± SEM. n = 3. ^#^ *p* < 0.05 compared with control, ^##^ *p* < 0.01 compared with control, *p < 0.05 compared with the OS-model group, **p < 0.01 compared with the OS-model group.

Insulin content is an essential indicator for evaluating pancreatic β cells function. Hence, we measured the level of insulin secretion of the β cells under EA protection. We employed a common model (high insulin secretion induced by treatment with KCl (30 mM) or low insulin secretion induced by treatment with glucose (5.5 mM)) (Yang et al., 2021) to investigate the effect of treatment with EA and alloxan or H_2_O_2_ upon insulin secretion by measuring insulin content in the supernatant of β cells using ELISAs. Insulin secretion decreased in oxidative stress-induced pancreatic β cells, but recovered under co-treatment with EA (Figures 4A, B). Flavonoids upregulate the protein expression (including PDX-1 expression) involved in pancreatic β cell function (Lee et al., 2021). Protein expression of PDX-1 increased markedly following EA treatment (Figures 4C, D). These results indicated that EA might stimulate insulin secretion by increasing PDX-1 expression. To test this hypothesis, we investigated the effects of EA treatment on basal glucose-stimulated insulin secretion of β cells. We treated INS-1 cells with EA alone and measured the mRNA expression of PDX-1 and insulin level. Surprisingly, the level of insulin of glucose-treated cells increased significantly upon EA treatment. Simultaneously, PDX-1 expression increased in an EA concentration-dependent manner (Figures 4E, F). Collectively, these results demonstrated that EA could protect β cells from apoptosis but also improve pancreatic β-cell function by activating insulin synthesis.

### 3.6 Bioactive components of EA

**FIGURE 5:**
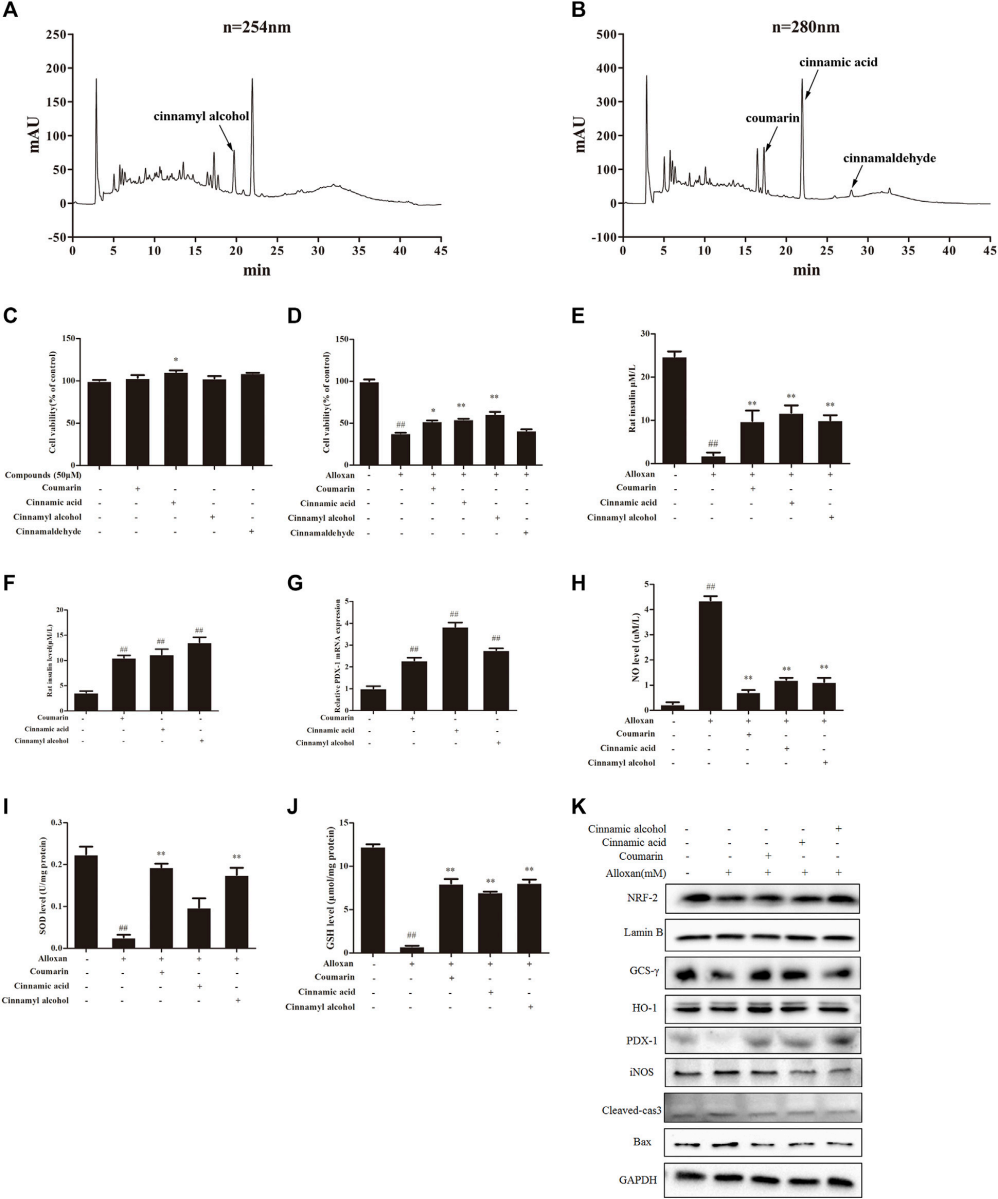
Major compounds of EA and their protective effect on β cells. Chemical profiles of EA were analyzed through HPLC and the effects of major functional compounds of EA on INS-1 cells were detected. **(A,B)** Chromatographic patterns from HPLC of EA shows peaks corresponding to retention times (min). INS-1 cells were incubated/not incubated with the main compounds (50 μM) of EA and alloxan for 24 h. The effect of compounds on the viability of INS-1 cells was determined by the CCK-8 assay **(C,D)**. The effect of three compounds (coumarin, cinnamic acid, cinnamic alcohol) in EA on KCl-stimulated insulin secretion in INS-1 cells under alloxan-induced toxicity **(E)**, basal glucose-stimulated insulin secretion in INS-1 cells **(F)**, mRNA expression of PDX1 in INS-1 cells **(G)**, NO release **(H)**, activity of SOD **(I)**, level of reduced GSH **(J)**, expression of antioxidant- and apoptosis-related proteins **(K)**. Data are the mean ± SEM. n = 3. ^#^ *p* < 0.05 compared with control, ^##^ *p* < 0.01 compared with control, *p < 0.05 compared with the OS-model group, **p < 0.01 compared with the OS-model group.

We carried out HPLC to elucidate the major functional compounds of EA: four major peaks appeared (Figures 5A, B). A previous study demonstrated that the chemical constituents of EA were primarily cinnamyl alcohol, coumarin, cinnamic acid, and cinnamaldehyde (Al-Dhubiab, 2012). Our HPLC results showed that EA mainly comprised (mg/g) coumarin (7.32), cinnamyl alcohol (7.87), cinnamic acid (4.37), and cinnamaldehyde (0.65). Therefore, we treated INS-1 cells and MIN6 cells with these four compounds individually (50 µM) for 24 h. Cell-viability assays showed that none of these four compounds had obvious toxicity towards β cells (Figure 5C). Treatment with coumarin, cinnamic acid, or cinnamic alcohol could recover the cell proliferation damaged by alloxan (Figure 5D). Moreover, these three compounds provoked an increase in KCl-stimulated insulin secretion compared with the OS-model group, which was similar to the effect of EA (Figure 5E). Coumarin, cinnamic acid, and cinnamic alcohol also increased basal glucose-stimulated insulin secretion in INS-1 cells. Then, the protein and mRNA expression of PDX1 was measured: treatment with any of these three compounds elicited a significant increase compared with that of the control (Figures 5F, G). To confirm the effect of coumarin, cinnamic acid, and cinnamic alcohol on alloxan- or H_2_O_2_ induced oxidative stress, the parameters of related markers (NO production, SOD activity, and level of reduced GSH) were measured. Similar to EA treatment, therapy with any of these three compounds suppressed NO release (Figures 5H–J), and maintained SOD activity and the GSH level. Furthermore, treatment with coumarin, cinnamic acid, or cinnamic alcohol increased expression of the antioxidant proteins Nrf2, γ-GCSc, and HO-1, while suppressing expression of the apoptosis-associated proteins iNOS, cleaved caspase 3, and BAX, in INS-1 cells treated with alloxan (Figure 5K). Notably, these three compounds promoted remarkable antioxidative capacity. Taken together, these data suggested that treatment with coumarin, cinnamic acid, or cinnamic alcohol from EA had an obvious protective effect on oxidative stress-induced β cell damage.

## 4 Discussion

T2DM is influenced primarily by the eating habits and sedentary lifestyles of some people. It is characterized by disorders of the metabolism of glucose and lipids, with deactivation of the insulin signaling pathway and abnormally increased blood glucose levels (Kahn, 2003). Pancreatic β cells are crucial for maintaining glucose homeostasis in the body by generating insulin. Studies have indicated that various extracts of cinnamon (ether, aqueous, methanolic) could be antioxidants (Mancini-Filho et al., 1998). Different flavonoids isolated from cinnamon have free radical-scavenging activities (Okawa et al., 2001). We discovered that EA constituents from cinnamon were rich in flavonoids. We demonstrated that EA has antioxidant activity in two models.

Hyperglycemia promotes oxidative stress (Bravard et al., 2011; Choudhuri et al., 2013), which causes the oxidation of lipids, proteins, and DNA, thereby leading to β cell damage. Enhanced oxidative stress plays a crucial part in DM pathogenesis. It contributes to cellular damage by increasing levels of oxidative-stress markers and reducing antioxidant levels in rodents and patients suffering from DM (Čater and Krizancic Bombek, 2022). Therefore, inhibiting ROS is important for preventing DM. It has been reported that *Cinnamomum zeylanicum* extracts reduced Aβ(1–42)-induced ROS production (Althobaiti et al., 2022). Recently, flavonoid compounds have attracted great interest for their potential use in DM treatment due to their remarkable antioxidant capacity. The proposed mechanism of flavonoids on antioxidant or anti-inflammatory activities might be: reducing ROS levels; activating anti-apoptosis pathways; inhibiting generation of NO (Ghorbani et al., 2019). We showed that EA from cinnamon reduced ROS production significantly in damaged β cells.

The MDA level can reflect the level of lipid-associated ROS. There are two types of antioxidant systems in the body: enzyme antioxidant system (including SOD) and the non-enzymatic antioxidant system (including GSH). In the present study, a significant increase in ROS accumulation and remarkable reductions in levels of antioxidative enzymes (e.g., SOD, reduced GSH) were observed in INS-1 cells and MIN6 cells after exposure to oxidative stress induced by alloxan or H_2_O_2_. Nonetheless, these oxidative stress-induced cellular events were blocked to a great extent when β cells were co-incubated with EA. These results suggested that enhancement of the endogenous antioxidant system, inhibition of intracellular release of ROS, and attenuation of lipid peroxidation may represent important mechanisms of cellular protection elicited by EA, which might be due mainly to its chemical characteristics. A growing body of work has indicated that activation of Nrf2 could regulate redox homeostasis, thus blocking DM pathogenesis (Behl et al., 2021). Our results demonstrated that EA treatment activated Nrf2 and its downstream cytoprotective genes (HO-1, γ-GCSc), which were verified to be Nrf2 activators. Hence, activating Nrf2 by EA could protect and activate β cells.

A simultaneous increase in ROS accumulation is associated with an increase in NO production. Excessive and sustained generation of NO derived from iNOS plays an important part in induction of β cell apoptosis in DM pathogenesis (Wang et al., 2014). A previous study found that cinnamaldehyde could prevent NO production and iNOS expression (Lee et al., 2002). We found that EA could significantly decrease the production of NO and expression of iNOS induced by treatment with alloxan or H_2_O_2_. In addition, it has been suggested that an increase in the level of pro-apoptotic proteins is associated with apoptosis. To further elucidate the anti-apoptosis effect of EA, we explored the possible effect of EA treatment on the activity of caspase-3 and caspase-9, as well as expression of cleaved caspase-3 and Bax. We demonstrated that EA treatment downregulated expression of iNOS. Our results further support the notion that EA has a pivotal anti-apoptotic role in DM.

It is believed that PDX-1 is an important target in the fight against DM. There is evidence that EA has significant protective effects upon β cell viability, possibly due to a shielding effect on insulin synthesis. PDX-1 has been reported to control the secretion of insulin as well as other β cell-specific genes. As expected, we found that EA treatment increased PDX-1 expression and insulin secretion, which implied that EA increases insulin synthesis in β cells. The beneficial effect of EA on promoting PDX-1 expression needs further investigation.

Our study suggested that EA has a robust protective role against damage of β cells, so we explored which monomer in EA is responsible. HPLC results showed the more abundant monomers are cinnamyl alcohol, coumarin, cinnamic acid, and cinnamaldehyde, they are phenylpropanoids. Further study revealed coumarin, cinnamic acid, and cinnamyl alcohol had protective effects upon β cells. Another compound from coumarin, scopoletin, has been reported to regulate glucose metabolism by enhancing the activities of antioxidant enzymes (Kalpana et al., 2019). In addition, cinnamic acid isolated from the hydro-alcohol extract of *Cinnamomum cassia* has been found to activate glucose transport in L6 myotubes through involvement of glucose transporter-4 *via* a phosphoinositide 3-kinase-independent pathway (Sangeetha et al., 2017). In a related study, cinnamyl alcohols were found to interact with the peroxisome proliferator-activated receptor gamma receptor, which is a key regulator of the metabolism and storage of lipids and glucose (Genovese et al., 2021). The studies mentioned above indicated that coumarin, cinnamic acid, and cinnamyl alcohol could have protective roles in DM, and our results provide more detailed explanations for these function. Future studies should focus on the detailed mechanism of action of these three monomers. Moreover, the chemical composition of cinnamon is relatively complex, whether the analogs of these ingredients in cinnamon also have similar activity warrants investigation. Our results in Table1 showed that the EA is rich in flavonoids, and we also found many small peaks in the HPLC analysis, which might be flavonoids with variety and low content. The flavonoids in this extract will be identified through UHPLC-MS/MS and further to elucidate the effect of cinnamon on antidiabetic activity in the next study.

We demonstrated the antioxidant function and possible mechanism of action of EA and the main compounds from cinnamon using two cell lines, it is still necessary to confirm our findings with *in vivo* research. Although many natural antioxidants from plants such as phenylpropanoids and flavonoids have been shown to be helpful to human health from different perspectives, their low solubility, poor absorption, rapid metabolism, and low bioavailability limit their clinical application (Teng et al., 2023). Hence, further structural modifications and technological approaches are needed to overcome this issue (Teng et al., 2021; Teng et al., 2021; Lyu et al., 2022).

## 5 Conclusion

Our study explained how EA protects β-cell from oxidative stress damage and improves β-cell function. EA treatment could prevent oxidative stress-induced apoptosis by blocking ROS accumulation and enhancing antioxidant capacities *via* activation of the Nrf2/HO-1 signaling pathway in two pancreatic β cell line models. EA might also be able to increase PDX-1 expression and insulin secretion. This study contributes to deeper understanding of the molecular and biological mechanisms of EA from CCE and its anti-DM functions.

## Data Availability

The original contributions presented in the study are included in the article/supplementary material, further inquiries can be directed to the corresponding authors.

## Abbreviations

CCE, Cinnamon cortex extract; CCK-8, Cell Counting Kit-8; DCFH-DA, 2′,7′-dichlorofluoresceindiacetate; DM, Diabetes mellitus; DMEM, Dulbecco’s modified Eagle’s medium; EA, ethyl acetate fraction; ELISA, Enzyme-linked immunosorbent assay; GSH, reduced glutathione; GCS-γ, gamma glutamylcysteine synthetase; HBSS, Hank’s balanced salt solution; HO-1, heme oxygenase 1; iNOS, inducible nitric oxide synthase; MDA, Malondialdehyde; NO, Nitric oxide; Nrf2, nuclear factor erythroid 2 related factor 2; PVDF, polyvinylidene fluoride; PBS, phosphate-buffered saline; ROS, Reactive oxygen species; SOD, Superoxide dismutase; VC, vitamin C.
